# Nomogram and risk calculator for severe hypoxemia after heart valve surgery

**DOI:** 10.3389/fcvm.2022.972449

**Published:** 2022-08-04

**Authors:** Xiangchao Ding, Dan Cheng, Bing Sun, Manda Sun, Chuangyan Wu, Jiuling Chen, Xiaoli Li, Yuan Lei, Yunshu Su

**Affiliations:** ^1^Department of Thoracic Surgery, Renmin Hospital of Wuhan University, Wuhan, China; ^2^Department of Cardiovascular Surgery, Union Hospital, Tongji Medical College, Huazhong University of Science and Technology, Wuhan, China; ^3^Department of Respiratory and Critical Care Medicine, Renmin Hospital of Wuhan University, Wuhan, China; ^4^Wuhan Third Hospital (Tongren Hospital of Wuhan University), Wuhan, China; ^5^Department of Pharmaceutical Biotechnology, The Queen's University of Belfast Joint College, China Medical University, Shenyang, China; ^6^Department of Thoracic Surgery, Union Hospital, Tongji Medical College, Huazhong University of Science and Technology, Wuhan, China; ^7^Department of Respiratory and Critical Care Medicine, Dawu County Hospital of Traditional Chinese Medicine, Xiaogan, China; ^8^Department of Gerontology, Renmin Hospital of Wuhan University, Wuhan, China

**Keywords:** hypoxemia, heart valve surgery, risk factor, nomogram, risk calculator

## Abstract

**Background:**

Hypoxemia is a very common issue in patients undergoing heart valve surgery (HVS), related to poor clinical outcomes. However, studies on severe hypoxemia (SH) after HVS have not been reported. The aims of this study were to identify predictors for SH in patients undergoing HVS and to develop and validate a risk prediction model.

**Methods:**

Patients undergoing HVS between 2016 and 2019 in a cardiovascular center were enrolled and were assigned to training and validation sets by a 7:3 ratio. Based on whether patients developed SH, they were divided into two groups. By univariate and multivariate analysis, predictors for SH were identified. Based on the predictors and logistic rule, a nomogram and a risk calculator were generated. The model was evaluated using calibration, discrimination and clinical utility.

**Results:**

The incidence rates of SH, moderate hypoxemia and mild hypoxemia were respectively 2.4, 23.9, and 58.2%. By multivariate analysis, seven independent risk factors for SH after HVS were identified, including body mass index, chronic obstructive pulmonary disease, renal insufficiency, white blood cell count, serum globulin, cardiopulmonary bypass time, and surgical types. The logistic model demonstrated satisfactory discrimination, calibration and clinical utility in both the training and validation sets. A nomogram and a risk calculator based on the logistic model were generated for easy application. Risk stratification was performed and three risk intervals were defined according to the nomogram and clinical practice. In addition, compared to patients without SH, patients with SH had significantly poorer clinical outcomes.

**Conclusions:**

Postoperative hypoxemia was prevalent after HVS, related to poor clinical outcomes. A logistic model including seven independent predictors for SH after HVS were established and validated, which demonstrated satisfactory discrimination, calibration and clinical utility. The results of this study may provide help to individualized risk assessment, early prevention and perioperative management.

## Introduction

As one of the most prevalent respiratory events after heart valve surgery (HVS), postoperative hypoxemia is associated with higher risks of poorer clinical outcomes (1–3). The incidence of hypoxemia varies extensively in different studies depending on different definitions and populations sampled, most ranging from 20 to 50% (3–6).

Studies focused on severe hypoxemia (SH) have been conducted in various surgical procedures, especially in acute aortic dissection surgery due to the ultrahigh prevalence (1, 4, 5, 7, 8). Some independent risk factors for postoperative SH have been identified and several prediction models have been constructed in previous studies (3, 4, 8, 9). However, most of these previous studies were based on small sample sizes and lacked statistical power, which may limit the clinical utility. Moreover, some studies were conducted decades ago and may not be suitable for current modern clinical applications. In addition, none of these previous studies were conducted in patients undergoing HVS. Predictors for SH after HVS remain to be explored and establishment of a risk prediction model is an urgent priority.

The aims of this study were first to elucidate the incidence and predictors of SH after HVS and to develop and validate a risk prediction model, and second to elucidate the impact of SH on clinical outcomes.

## Materials and methods

### Ethical statement

This study was carried out in accordance with the Declaration of Helsinki's ethical principles, and the Ethics Committee of Renmin Hospital of Wuhan University approved this study. The requirement for written informed consent was waived because of the retrospective and observational nature.

### Study population

This was a single-center, retrospective, observational study. Consecutive adult patients who underwent HVS in a tertiary cardiovascular center from 2016 to 2019 were identified. Patients with one or more of the following conditions were excluded from the current study: (1) age <18 years; (2) severe adverse respiratory or cardiovascular events and mechanical ventilation within 2 weeks before surgery; (3) history of immunodeficiency, immunosuppression, and organ transplant; (4) intraoperative or early postoperative death; and (5) incomplete medical records.

### Data collection

We conducted a comprehensive data collection using the hospital's electronic medical records system, including pre-, intra-, and post-operative variables. Preoperative variables included demographics, underlying conditions, comorbidities, ultrasound results, and laboratory values. Intraoperative factors included surgical types, cardiopulmonary bypass (CPB) time, aortic cross clamp time, and transfusion of red blood cells (RBCs). Postoperative variables included the durations of mechanical ventilation, intensive care unit (ICU) stay and hospital stay, the rates of pneumonia, readmission to ICU, reintubation, tracheostomy, and in-hospital death.

### Endpoints and definitions

Postoperative arterial blood gases were routinely measured using blood gas analyzers (Radiometer, ABL800PLEX, Denmark). The ratios of arterial oxygen tension to inspired oxygen concentration (PaO_2_/FiO_2_) were calculated. According to the widely accepted Berlin definition for acute respiratory distress syndrome and previous reports in the literature, we defined mild hypoxemia as 200 mmHg < PaO_2_/FiO_2_ ≤ 300 mmHg, moderate hypoxemia as 100 mmHg < PaO_2_/FiO_2_ ≤ 200 mmHg, and SH as PaO_2_/FiO_2_ ≤ 100 mmHg (1, 10). The primary endpoint of this study was SH after HVS within the first 24 h postoperatively, and all the patients were classified into two groups based on whether SH developed.

### Statistical analysis

R software (version 4.0.5) and IBM SPSS (version 26.0) were used to conduct statistical analysis. Two-tailed *P* < 0.05 were considered to be statistically significant.

Patients were randomly assigned to training and validation sets by 7:3 ratio. Categorical variables were presented as numbers and proportions. Normally distributed continuous variables were presented as means and standard deviations. Non-normally distributed continuous variables were presented as medians and ranges. Kolmogorov-Smirnov tests were used to examine whether continuous variables were normally distributed. For univariate analysis, Fisher's exact test or chi-square test were applied to categorical variables, Student's *t*-test were applied to normally distributed continuous variables with variance homogeneity, and Mann-Whitney U-test were applied otherwise. Factors with *P* < 0.25 in the univariate analysis or considered clinically significant were further analyzed by multivariate logistic regression analysis using a forward stepwise procedure to identify independent risk factors for SH after HVS. The coefficient, standard error, odds ratio (OR) with 95% confidence interval (CI), and P value of each predictor was calculated and presented. Based on these predictors and logistic rule, a graphical nomogram and a web-based online risk calculator were constructed.

Internal validation was completed using bootstrap method with 1,000 replications. External validation was completed in the independent validation set. Calibration was assessed by calibration plot and Hosmer-Lemeshow goodness-of-fit test. Discrimination was assessed by the area under the receiver operating characteristic (ROC) curve (AUC). The comparison of the two AUCs was completed by Delong method (11). Clinical utility was assessed by decision curve analysis.

## Results

### Demographic characteristics

A total of 3,853 patients fulfilled the inclusion criteria and were analyzed in this study, 54% were males and 46% were females. The mean age of these included patients was 51 years, with a mean BMI of 23 kg/m^2^. The incidence rates of mild hypoxemia, moderate hypoxemia, and SH after HVS were respectively 58.2, 23.9, and 2.4%.

Multiple underlying conditions and comorbidities were present in this study population. Patients with a smoking history accounted for 26.7%, drinking history for 20.1%, hypertension for 24.2%, diabetes mellitus for 5.7%, chronic obstructive pulmonary disease (COPD) for 12.9%, atrial fibrillation for 23.3%, renal insufficiency for 8.2%, pulmonary edema for 6.0%, pulmonary artery hypertension for 32.1%, pericardial effusion for 15.6%, gastrointestinal tract disease for 8.2%, cardiac surgery history for 8.0%, and general surgery history for 29.7%.

Three-quarters of the operations were isolated valve surgery, 13% had concomitant coronary artery bypass grafting (CABG), 11% had concomitant aortic surgery, and 2% had concomitant CABG as well as aortic surgery. The median durations of CPB and aortic cross clamp were respectively 108 (86, 139) and 72 (54, 95) min, and the volume of transfused RBC was 1 (1, 3) units. The incidence rate of SH was 1.2% in isolated valve surgery, 2.7% in concomitant CABG, 7.0% in concomitant aortic surgery, and 20.6% in concomitant CABG and aortic surgery.

### Model development

**Figure 1:**
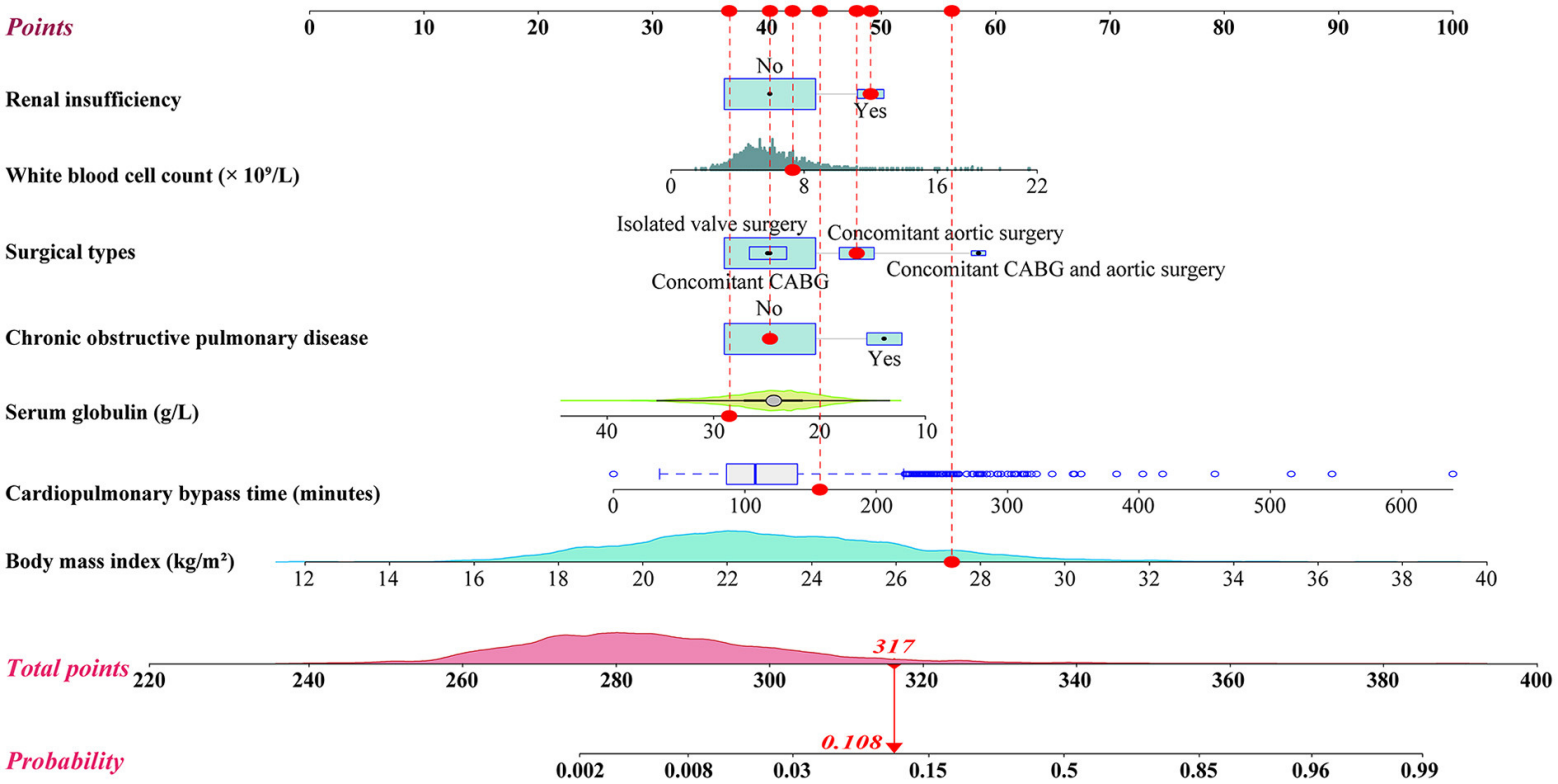
Nomogram for the prediction of severe hypoxemia after heart valve surgery.

We initially conducted univariate analysis to screen potential risk factors for SH after HVS in the training set (Table 1). Factors with *P* < 0.25 or considered clinically significant were further analyzed by multivariate logistic regression analysis, including sex, BMI, smoking history, drinking history, hypertension, COPD, atrial fibrillation, renal insufficiency, pulmonary edema, general surgery history, pulmonary artery hypertension, pericardial effusion, diameters of the left and right atrium, left ventricular ejection fraction, white blood cell count (WBC) count, hemoglobin, serum albumin, globulin, creatinine, surgical types, CPB time, and transfusion of RBCs. Seven independent risk factors were finally identified in the multivariate analysis, including surgical types, body mass index (BMI), CPOD, renal insufficiency, WBC count, serum globulin, and CPB time (Table 2). A logistic regression model were constructed using the seven predictors above. A nomogram and a web-based online risk calculator were then generated based on the multivariate logistic model (Figure 1, https://hypoxemiaafteHVS.shinyapps.io/dynnomapp/).

**Table 1:** Univariate analysis of possible risk factors for SH following HVS in the training set.

**Characteristics**	**Without SH**	**With SH**	**χ^2^/Z/t**	***P* value**
	***n* = 2,634 (%)**	***n* = 63 (%)**		
**Demographics**				
Male	1,405 (53.3)	49 (77.8)	14.787	<0.001
Age (years)	51.15 ± 12.48	52.46 ± 10.70	0.824	0.410
Body mass index (kg/m^2^)	22.91 ± 3.21	27.22 ± 4.03	8.415	<0.001
Smoking history	696 (26.4)	32 (50.8)	18.543	<0.001
Drinking history	529 (20.1)	26 (41.3)	16.898	<0.001
**Underlying conditions**				
Hypertension	599 (22.7)	37 (58.7)	44.223	<0.001
Diabetes mellitus	142 (5.4)	5 (7.9)	0.774	0.379
Chronic obstructive pulmonary disease	337 (12.8)	12 (19.0)	2.136	0.144
Cerebrovascular disease	917 (34.8)	22 (34.9)	0.001	0.986
Peripheral vascular disease	1,104 (41.9)	24 (38.1)	0.369	0.544
Atrial fibrillation	628 (23.8)	8 (12.7)	4.240	0.039
Renal insufficiency	189 (7.2)	21 (33.3)	58.634	<0.001
Gastrointestinal tract disease	210 (8.0)	5 (7.9)	<0.001	0.992
Pulmonary edema	152 (5.8)	7 (11.1)	3.163	0.075
Cardiac surgery history	202 (7.7)	5 (7.9)	0.006	0.937
General surgery history	786 (29.8)	12 (19.0)	3.440	0.064
NYHA class III-IV	488 (18.5)	11 (17.5)	0.046	0.829
Pulmonary artery hypertension	855 (32.5)	14 (22.2)	2.953	0.086
Pericardial effusion	412 (15.6)	15 (23.8)	3.080	0.079
Diameter of the left atrium (cm)	4.5 (3.9, 5.3)	4.4 (3.7, 5.1)	0.757	0.449
Diameter of the left ventricle (cm)	5.3 (4.6, 6.0)	4.8 (5.5, 6.2)	1.409	0.159
Diameter of the right atrium (cm)	3.9 (3.5, 4.5)	4.0 (3.6, 4.4)	0.755	0.450
Diameter of the right ventricle (cm)	3.6 (3.3, 4.0)	3.8 (3.5, 4.1)	2.007	0.045
Left ventricular ejection fraction (%)	62 (58, 66)	60 (59, 64)	1.384	0.166
**Laboratory values**				
White blood cell count (×10^9^/L)	5.56 (4.61, 6.79)	7.86 (5.96, 9.95)	6.674	<0.001
Red blood cell count (×10^12^/L)	4.27 (3.92, 4.63)	4.42 (4.19, 4.78)	2.620	0.009
Hemoglobin (g/l)	129 (118, 141)	126 (137, 146)	3.288	0.001
Platelet count (×10^9^/L)	175 (141, 214)	159 (128, 215)	1.215	0.224
Serum albumin (g/L)	40.4 (38.0, 42.7)	39.7 (37.2, 42.1)	1.495	0.135
Serum globulin (g/L)	24.3 (21.6, 27.1)	24.2 (21.5, 25.8)	1.152	0.249
Serum creatinine (μmol/L)	71.2 (60.9, 84.0)	79.4 (71.1, 95.1)	3.881	<0.001
**Surgical types**			96.514	<0.001
Isolated valve surgery	1,991 (75.6)	24 (38.1)		
Concomitant CABG	329 (12.5)	9 (14.3)		
Concomitant aortic surgery	274 (10.4)	21 (33.3)		
Concomitant CABG and aortic surgery	40 (1.5)	9 (14.3)		
CPB time (min)	108 (86, 139)	191 (125, 261)	8.334	<0.001
Aortic cross clamp time (min)	72 (54, 95)	112 (81, 153)	6.975	<0.001
Transfusion of red blood cells (units)	1 (1, 3)	3 (1, 7)	4.630	<0.001

*CABG, coronary artery bypass graft; HVS, heart valve surgery; NYHA, New York Heart Association; SH, severe hypoxemia*.

**Table 2:** Multivariate analysis of independent risk factors for SH following HVS.

**Characteristic**	**Coefficient**	**Standard error**	**OR (95% CI)**	***P* value**
Surgical types				0.010
Isolated valve surgery	Reference	Reference	Reference	Reference
Concomitant CABG	−0.017	0.444	0.983 (0.412–2.344)	0.969
Concomitant aortic surgery	0.746	0.411	2.109 (0.942–4.722)	0.070
Concomitant CABG and aortic surgery	1.796	0.57	6.026 (1.961–18.514)	0.002
Body mass index (kg/m^2^)	0.363	0.043	1.438 (1.322–1.565)	<0.001
Chronic obstructive pulmonary disease	0.984	0.376	2.676 (1.282–5.586)	0.009
Renal insufficiency	0.867	0.356	2.381 (1.185–4.782)	0.015
White blood cell count (×10^9^/L)	0.143	0.052	1.154 (1.042–1.279)	0.006
Serum globulin (g/L)	−0.091	0.039	0.913 (0.846–0.985)	0.019
Cardiopulmonary bypass time (min)	0.011	0.002	1.011 (1.007–1.015)	<0.001
Intercept	−13.878	1.509	<0.001	<0.001

*CABG, coronary artery bypass graft; CI, confidence interval; HVS, heart valve surgery; OR, odds ratio; SH, severe hypoxemia*.

### Model validation and assessment

**Figure 2:**
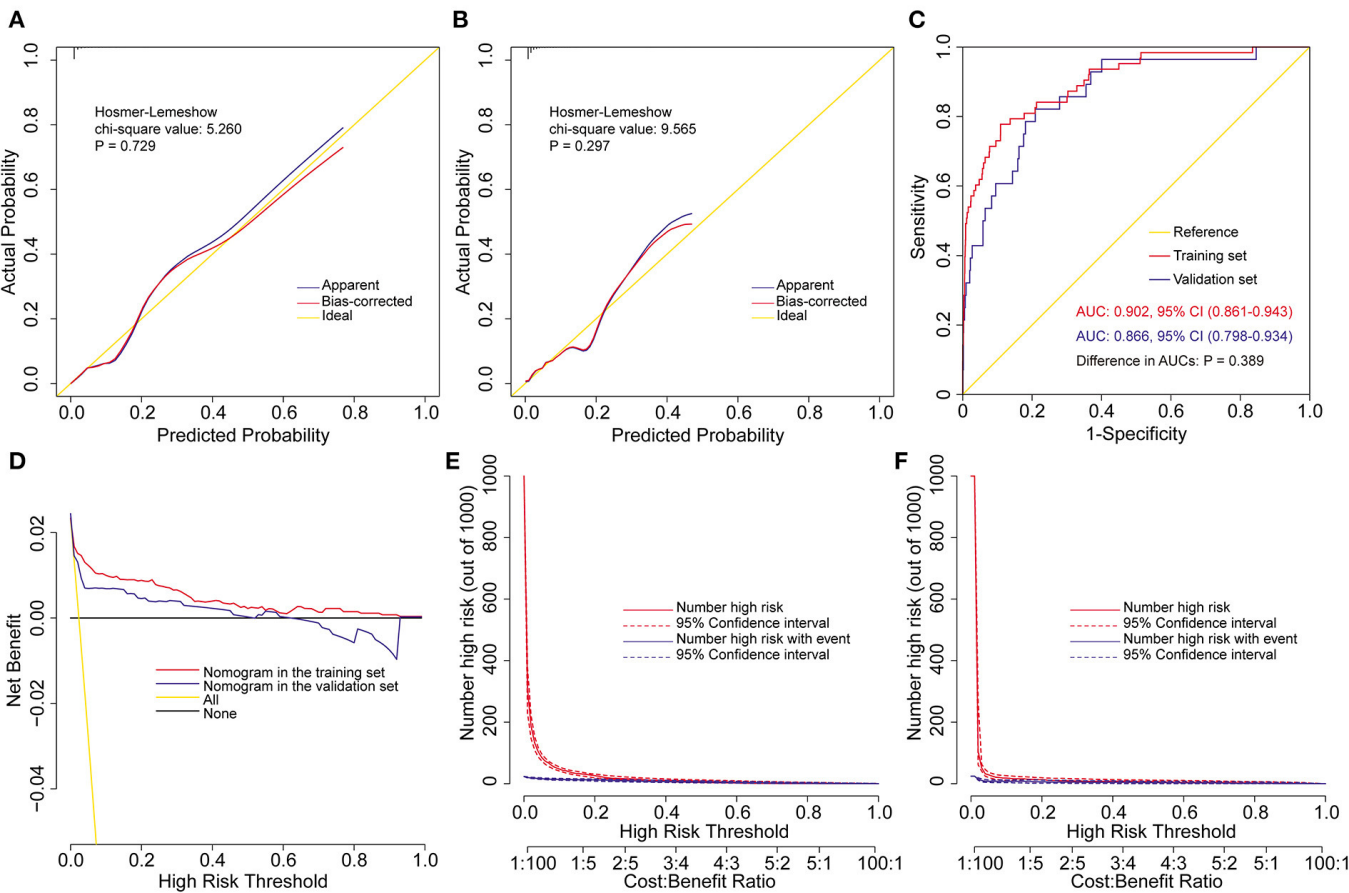
Assessment of the prediction model for severe hypoxemia after heart valve surgery. Calibration plots and goodness-of-fit test in the training set **(A)** and the validation set **(B)**, ROC curves and the AUC in the training and validation sets **(C)**, decision curves in the training and validation sets **(D)**, and clinical impact curves in the training set **(E)** and the validation set **(F)**. AUC, area under the receiver operating characteristic curve; CI, confidence interval; ROC, receiver operating characteristic curve.

The model was well validated internally in the training set and externally in the independent validation set. By visual inspection of the calibration curves in the two sets, the model showed good agreement between prediction and observation (Figures 2A,B). The Hosmer-Lemershow goodness-of-fit test also confirmed the consistency of fit of the model, with a chi-square value of 5.260 (P = 0.729) in the training set and 9.565 (P = 0.297) in the validation set. The AUC of the model was 0.902 (95% CI: 0.861–0.943) in the training set and 0.866 (95% CI: 0.798–0.934) in the validation set, which demonstrated excellent discrimination (Figure 2C). The difference between the two AUCs were not statistically different. The optimal cutoff of predicted probability was 0.032, with sensitivity, specificity, positive predictive value and negative predictive value of 77.8, 89.1, 14.6, and 99.4%, respectively. The decision and clinical impact curves showed that the model had good clinical utility (Figures 2D–F). More clinical net benefits could be obtained compared to the “treat-all/none” strategies among all threshold probabilities in the training set and among the thresholds of below 0.6 in the validation set.

### Risk stratification

We further conducted a risk stratification for SH after HVS to facilitate clinical application based on the nomogram model and clinical practice (Table 3). All the patients were stratified into three risk intervals named low-, medium-, and high-risk groups, corresponding to scores of <292, 292–308, and >308 points on the nomogram, with cutoff values of the predicted probabilities of 0.01 and 0.05. The observed and predicted probabilities within each risk group showed good consistency. The differences among the three risk groups were significant, which showed the rationality of the risk division. In this study, 69.4% of the patients were divided into the low-risk group, 21.9% into the medium-risk group, and 8.7% into the high-risk group.

**Table 3:** Risk intervals of SH based on the nomogram and clinical practice.

**Risk intervals**	**Low risk**	**Medium risk**	**High risk**
	**(<292 points)**	**(292–308 points)**	**(>308 points)**
Estimated probability (%)	<1	1–5	>5
Estimated probability, % (95% CI)	0.32 (0.31–0.33)	2.17 (2.10–2.24)	19.02 (17.03–21.00)
Observed probability, % (95% CI)	0.52 (0.25–0.80)	2.25 (1.25–3.25)	17.26 (13.20–21.32)
No. of patients (%)	2,673 (69.4)	844 (21.9)	336 (8.7)

*CI, confidence interval; SH, severe hypoxemia*.

### Clinical outcomes

There was an overall mortality rate of 2.9% (111/3,853) among the included patients, with a rate of 16.5% in patients with SH vs. 2.6% in patients without SH (P < 0.001). In addition, the rates of postoperative pneumonia, readmission to ICU, reintubation and tracheostomy were significantly higher in patients with SH, and the mechanical ventilation, ICU and hospital stay were significantly prolonged compared to patients without SH. Details of the comparison of these outcomes are listed in Table 4.

**Table 4:** Postoperative variables in patients with and without SH following HVS.

**Variables**	**Without SH**	**With SH**	**χ^2^/Z**	***P* value**
	***n* = 3,762 (%)**	***n* = 91 (%)**		
Mechanical ventilation (h)	22.2 (18.7, 41.5)	71.9 (44.0, 165.4)	11.164	<0.001
Pneumonia	278 (7.4)	36 (39.6)	122.849	<0.001
Reintubation	143 (3.8)	12 (13.2)	20.272	<0.001
Tracheostomy	56 (1.5)	12 (13.2)	70.134	<0.001
Readmission to ICU	131 (3.5)	14 (15.4)	34.755	<0.001
ICU stay (h)	67.3 (44.6, 95.8)	163.2 (111.3, 285.4)	11.307	<0.001
Hospital stay (days)	14 (11, 19)	22 (16, 31)	7.590	<0.001
Mortality	96 (2.6)	15 (16.5)	61.637	<0.001

*HVS, heart valve surgery; ICU, intensive care unit; SH, severe hypoxemia*.

## Discussion

Postoperative hypoxemia has been reported to be prevalent and related to increased risk of various poor prognosis after multiple surgeries (1, 2, 12), which was consistent with the results of the current study. In this study, the overall incidence rates of mild hypoxemia, moderate hypoxemia, and SH after HVS were respectively 58.2, 23.9, and 2.4%. The overall mortality rate was 2.9%, and the mortality rates in patients without hypoxemia, with mild hypoxemia, moderate hypoxemia and SH were respectively 1.7, 2.2, 4.7, and 16.5%. In addition, the risks of multiple poor clinical outcomes were also significantly increased in patients with SH, which stressed the need of identifying independent risk factors for SH after HVS and developing as well as validating a convincing risk prediction model.

In this study, we used clinical data from 3,853 adult patients undergoing HVS at a single institution to explore significant predictors for SH and to develop and validate a risk prediction model. After multivariate logistic regression analysis, seven factors were identified to significantly associate with the development of SH after HVS, including BMI, CPOD, renal insufficiency, WBC count, serum globulin, surgical types, and CPB time. A visual nomogram and a web-based online risk calculator used to predict the risk of SH after HVS were then generated based on the logistic regression model. The model indicated excellent discrimination, calibration and clinical utility, and was well validated externally in the independent dataset. Based on the nomogram and clinical practice, three risk groups were divided as low-, medium-, and high-risk groups. To our knowledge, our work represents the first attempt to develop and validate a nomogram model and the first web-based online risk calculator for SH after HVS worldwide, which may be clinically instructive.

Numerous reports have been published regarding postoperative hypoxemia due to its prevalence in various operations (1, 4, 5, 7, 8), however, none of these previous studies were conducted in patients undergoing HVS. Several predictors for postoperative hypoxemia have been reported in the literature, among which obesity was the most frequently reported one (4, 6, 9, 12–16), which was in agreement with our results. In this study, the risk of SH after HVS increased significantly with BMI, and BMI showed the greatest contribution in predicting SH among all those predictors. Gong et al. conducted a single-center retrospective study in patients undergoing urgent aortic arch surgery for acute type A aortic dissection to identify the risk factors for SH, finding that increased BMI was an independent predictor, with an OR value of 1.47 (95% CI: 1.21–1.79, *P* < 0.001) (4). They believed that this was associated with the decrease in lung compliance, the increase in respiratory resistance and breathing difficulties among the obese. Another retrospective study conducted in patients with acute aortic dissection by Ge et al. found that BMI was significantly related to the increase of postoperative hypoxemia and was included in their final nomogram model, with an OR value of 1.32 (95% CI: 1.15–1.54, *P* < 0.001) (8). They believed that this could be explained by the fact that BMI was an important determinant of respiratory function and patients with higher BMI may have a typical restrictive pattern with a reduction of forced residual capacity, forced vital capacity and total lung capacity. Furthermore, recent studies have suggested that oxidative stress and inflammatory response was associated with the process of lung injury caused by obesity, which provided new directions for future research (9).

Another independent preoperative risk factors for SH after HVS identified in our analysis was WBC count, which was also widely reported in the literature (5, 8). A retrospective study conducted by Liu et al. reported that higher WBC count was independently related to higher risk of postoperative hypoxemia in patients undergoing surgery for acute aortic dissection, and hypoxemia was significantly related to longer mechanical ventilation, prolonged ICU and hospital stay (5). Ge et al. obtained similar results, who reported that WBC count was positively associated with postoperative hypoxemia and included WBC into their final regression model for postoperative hypoxemia after acute aortic dissection surgery (8). A systemic inflammatory response may explain to some degree the relationship between elevated WBC and hypoxemia. High WBC counts are important biomarkers because they indicate more intense inflammatory responses that may lead to respiratory dysfunction and hypoxemia (17, 18). C-reactive protein levels and interleukin-6 have also been reported to have a correlation with hypoxemia in previous studies, which have been identified as nonspecific and sensitive markers of acute phase reactions (17). The relationship between hypoxemia and inflammatory response has received considerable attention recently, providing new and enlightening perspectives (19). Additionally, the use of some medications like ulinastatin have been reported to provide significant protection (17).

COPD was another significant preoperative predictor for SH after HVS identified in our analysis, similar to some of the previous reports (20, 21). Liu et al. reported that COPD was one of the independent risk factors for hypoxemia during minithoracotomy direct-vision CABG, with a 3.4-fold increased risk (21). They believed that patients with COPD may have limited ability to provide sufficient oxygen to maintain adequate arterial oxygenation during surgery because intrapulmonary shunting was elevated when mechanically ventilated. Ji et al. reported that preoperative chronic pulmonary diseases was an independent risk factor for postoperative hypoxemia after decannulation following CABG, with an OR value of 7.19 (95% CI: 2.81–18.41, *P* < 0.001) in their multivariate analysis results (20). This may be partially explained by the fact that patients with chronic pulmonary diseases may have significantly reduced lung elastic recoil, narrower pulmonary airway, and loss of pulmonary capillary beds due to damaged pulmonary alveoli, resulting in ventilation/perfusion unbalance and gas exchange dysfunction.

Previous studies have also indicated that the duration of CPB was independently associated with the development of postoperative hypoxemia (8, 14), which was consistent with the results of this study. An observational transversal study designed to explore predictors for SH after myocardial revascularization reported that the use of CPB increased the risk of SH, and the odds of developing SH were 3.1 times greater in patients receiving CPB more than 120 min (14). Another retrospective study conducted in patients undergoing aortic dissection surgery reported that the duration of circulatory arrest was an independent predictor for postoperative hypoxemia (22). Liu et al. also conducted a retrospective study in patients undergoing surgical repair of acute type A aortic dissection to identify risk factors for hypoxemia, which found that the duration of deep hypothermic circulatory arrest longer than 25 min was independently associated with a 3.3-fold increased risk of postoperative hypoxemia (5). The internal mechanism between CPB and hypoxemia may be partially explained by the fact that CPB is a non-physiological circulation and the use of CPB can induce diverse inflammatory mediators and cytokines, result in systematic inflammatory responses, and weaken immune responses (23, 24). Although great improvement has been made in CPB technology over the past decades, prolonged use of CPB may significantly influence the perfusion of multiple organs and peripheral tissues. Recently, a concept of “more physiologic” cardiac surgery has been raised by clinical investigators and minimal invasive extracorporeal circulation has been introduced to overcome the deficiencies and weaknesses of traditional CPB (25, 26). Additionally, previous studies indicated that the experience of surgeons and the duration of the whole surgical procedures may also significantly contribute to the development of postoperative hypoxemia (7, 21). Thus, improved surgery skills and more training for surgeons are likely to benefit patients greatly.

The presence of renal insufficiency, the serum levels of globulin, and surgical types were also identified to associate with the development of SH after HVS in our analysis, which was in agreement with some previous reports in other surgeries (3, 7, 8). Ge et al. reported that patients undergoing surgery for type A aortic dissection had a 4.5-fold increased risk of postoperative hypoxemia compared to type B aortic dissection (8). Weiss and Rady et al. reported that emergency surgery correlated significantly with time to extubation and lung injury, and was independent predictor of early postoperative pulmonary dysfunction (27, 28). Previous studies have identified the close relationship between renal insufficiency and various postoperative complications after cardiovascular surgery, such as hypoxemia, pneumonia, hyperlactatemia, and tracheostomy (3, 29–33). Although the related researches on the relationship between renal insufficiency and hypoxemia are limited and the exact mechanisms remain to be further investigated, we guess that inflammatory responses, erythropoietin production by the kidney, and the subsequent oxygen delivery may be involved (34). Serum globulin levels were identified as a protective factor in our analysis, which may be related to the inflammatory status and the immune responses of the body.

In addition to the above mentioned risk factors for developing postoperative hypoxemia, several other risk factors have also been reported in the literature, including older age, female sex, smoking history, hypertension, low serum albumin, diabetes, poor cardiac function, and blood transfusion (1, 3, 7, 15, 16, 20–22, 28). The presence of postoperative hypoxemia has also been reported to be associated with some postoperative factors, however, we did not analyze or include these factors into the multivariate analysis due to the fact that these factors were not available early and we would fail to achieve the purpose of early prediction if these variables were included. Our findings suggested that the model constructed using only preoperative and intraoperative variables can perform well in various aspects.

Using the prediction model, we can accurately predict individual risk, identify high-risk groups and then prevent the development of adverse outcomes. Over the past few years, a series of measures have been suggested to prevent hypoxemia and acute respiratory distress syndrome, including prone positioning, lung recruitment maneuvers, positive end-expiratory pressure, ventilation with low tidal volume, high inspiratory pressure and high-frequency oscillation, and the application of extracorporeal membrane oxygenation (35). Providing proper preventive interventions and treatments to high-risk populations identified by our model can lead to considerable financial benefits and improved clinical outcomes.

There are some limitations in this study. First, this was a retrospective observational study conducted in only one cardiovascular center and was not validated using data from other centers, which may limit the generalizability of the model. Second, we failed to collect and include some possible risk factors into our analysis, such as preoperative PaO_2_/FiO_2_ and drug use. Third, we only studied the incidence, risk factors and related outcomes of hypoxemia within the first 24 h after surgery, and the overall situation of hypoxemia exceeding 24 hours postoperatively was not further analyzed. Fourth, we only collected and compared the clinical outcomes during hospitalization between patients with and without SH, but failed to conduct long-term follow-up after discharge, which should be strengthened in future studies.

## Conclusions

Hypoxemia was prevalent after HVS, portending poorer clinical outcomes. By multivariate analysis, seven independent risk factors for SH after HVS were identified, including surgical types, BMI, CPOD, renal insufficiency, WBC count, serum globulin, and CPB time. A nomogram and a risk calculator based on the logistic regression model were then generated, which indicated excellent discrimination, calibration, clinical utility, and was well validated. Three risk groups were divided as low, medium and high risk groups based on the nomogram and clinical practice. To our knowledge, this is the first attempt to develop and validate a nomogram model and the first web-based online risk calculator for SH after HVS worldwide, which may be helpful for early risk assessment and perioperative management.

## Data availability statement

The raw data supporting the conclusions of this article will be made available by the authors, without undue reservation.

## Ethics statement

The studies involving human participants were reviewed and approved by the Ethics Committee of Renmin Hospital of Wuhan University. Written informed consent for participation was not required for this study in accordance with the national legislation and the institutional requirements.

## Author contributions

YL, YS, and XD: conception and design. YS and DC: administrative support. BS and MS: provision of study materials or patients. CW and JC: collection and assembly of data. XL and XD: data analysis and interpretation. All authors: manuscript writing and final approval of manuscript.

## Funding

The work was supported by the China Scholarship Council Fund (201806275100), Hubei Key Laboratory of Wuhan University (2021KFY031), National Natural Science Foundation of China (81600023, 82100115, and 82100299), and Hubei Province Natural Science Foundation (2020CFB392).

## Conflict of interest

The authors declare that the research was conducted in the absence of any commercial or financial relationships that could be construed as a potential conflict of interest.

## Publisher's note

All claims expressed in this article are solely those of the authors and do not necessarily represent those of their affiliated organizations, or those of the publisher, the editors and the reviewers. Any product that may be evaluated in this article, or claim that may be made by its manufacturer, is not guaranteed or endorsed by the publisher.

## Abbreviations

AUC: area under the receiver operating characteristic curve
BMI: body mass index
CABG: coronary artery bypass grafting
CI: confidence interval
COPD: chronic obstructive pulmonary disease
CPB: cardiopulmonary bypass
HVS: heart valve surgery
ICU: intensive care unit
NYHA: New York Heart Association
OR: odds ratio
PaO_2_-FiO_2_: arterial oxygen tension-inspired oxygen concentration
RBC: red blood cell
ROC: receiver operating characteristic curve
SH: severe hypoxemia
WBC: white blood cell.
